# [Corrigendum] Effect of *STC2* gene silencing on colorectal cancer cells

**DOI:** 10.3892/mmr.2024.13386

**Published:** 2024-11-06

**Authors:** Qianyuan Li, Xiukou Zhou, Zhengyu Fang, Zhiyun Pan

Mol Med Rep 20: 977–984, 2019; DOI: 10.3892/mmr.2019.10332

Subsequently to the publication of the above paper, an interested reader drew to the authors’ attention that the ‘Control’ and ‘NC’ data panels shown in Fig. 2E on p. 981, showing the results of Transwell invasion assay experiments, appeared to contain overlapping sections of data, such that they were potentially derived from the same original source where these panels were intended to show the results from differently performed experiments. After having asked the authors to provide an explanation of these data, they realized that this figure had been inadvertently assembled incorrectly. A revised version of Fig. 2, containing replacement data for the experiments portrayed in Fig. 2E, is shown on the next page. Note that these errors did not adversely affect either the results or the overall conclusions reported in this study. All the authors agree with the publication of this corrigendum, and are grateful to the Editor of *Molecular Medicine Reports* for allowing them the opportunity to publish this. They also wish to apologize to the readership of the Journal for any inconvenience caused.

## Figures and Tables

**Figure 2.**
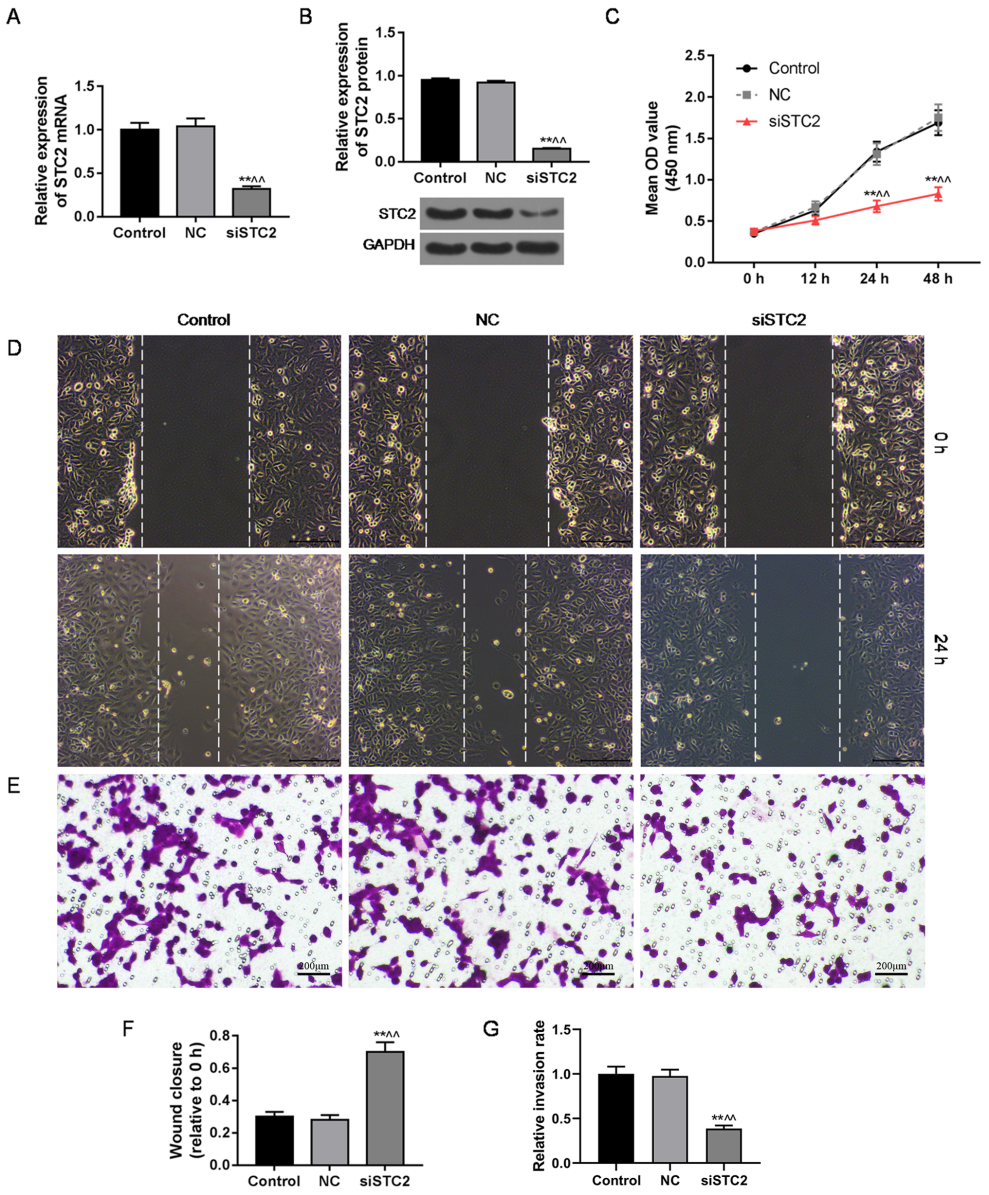
Silencing of STC2 displays an anticancer effect on CRC SW480 cells. (A) Quantitative real-time polymerase chain reaction (qPCR) and (B) western blot analysis were used to evaluate the efficiency of siSTC2 transfection. (C) The cell viability was identified by CCK-8 assay. The migration (D) and invasive (E) abilities were assessed by wound healing and Transwell assays. (F) The relative wound closure distance in the control, NC and siSTC2 groups. (G) The invasion rate of the control, NC and siSTC2 groups. All data are expressed as means ± SEM. **P<0.01 vs. control; ^^^^P<0.01 vs. NC. STC2, stanniocalcin 2; CCK-8, Cell Counting Kit-8..

